# Comparison of dual influenza and pneumococcal polysaccharide vaccination with influenza vaccination alone for preventing pneumonia and reducing mortality among the elderly: A meta-analysis

**DOI:** 10.1080/21645515.2016.1221552

**Published:** 2016-09-14

**Authors:** Yan-Yang Zhang, Xue-Feng Tang, Chang-Hui Du, Bin-Bing Wang, Zhen-Wang Bi, Bi-Rong Dong

**Affiliations:** a Henan Center for Disease Control and Prevention, Zhengzhou, Henan, China; b Sichuan Center for Disease Control and Prevention, Chengdu, Sichuan, China; c The Center of Gerontology and Geriatrics, West China Medical School/West China Hospital, Sichuan University, Chengdu, Sichuan, China; d Chengdu Center for Disease Control and Prevention, Chengdu, Sichuan, China; e Anhui Center for Disease Control and Prevention, Hefei, Anhui, China; f Institute of Bacterial Infectious Disease Control and Prevention, Shandong Center for Disease Control and Prevention, Jinan, Shandong, China

**Keywords:** aged, influenza vaccine, meta-analysis, mortality, pneumococcal vaccine, pneumonia

## Abstract

The purpose of this study was to perform a meta-analysis comparing the effectiveness of influenza vaccination alone versus influenza plus pneumococcal dual vaccination for the prevention of pneumonia and mortality in adults ≥ 65 years of age. Medline, Cochrane, CENTRAL, EMBASE, and Google Scholar databases were searched. Inclusion criteria were: 1) Randomized controlled trials (RCTs), 2-arm prospective studies, or retrospective cohort studies; 2) Patients were ≥ 65 years of age with or without chronic respiratory disease; 3) Patients received the influenza vaccine alone or dual pneumococcal and influenza vaccination; 4) Results included incidence of recurrent respiratory tract infections, length of hospital stay, and overall mortality rate. The outcomes were pneumonia and all-cause mortality rates. Of 142 studies identified in the database searches, 6 were ultimately included in the systematic review, and 5 were included in meta-analysis. The number of patients that received the influenza vaccination alone ranged from 211 to 29,346 (total = 53,107), and the number that received influenza+pneumococcal vaccination ranged from 246 to 72,107 (total = 102,068). Influenza+pneumococcal vaccination was associated with a significantly lower pneumonia rate than influenza vaccination alone (relative risk [RR] = 0.835, 95% confidence interval [CI]: 0.718–0.971, *P* = 0.019), and with a significantly lower all-cause mortality rate than influenza vaccination alone (relative risk [RR] = 0.771, 95% confidence interval [CI]: 0.707–0.842, *P* = 0.001). In conclusion, the results of this study support concomitant pneumococcal and influenza vaccination of the elderly as a dual vaccination strategy is associated with lower pneumonia and all-cause mortality rates.

## Introduction

Annual vaccination against influenza is recommended for the elderly by the World Health Organization. However, increasing evidence suggests that available influenza vaccines are less effective in the elderly compared to younger adults.^1^ Vaccine effectiveness estimates vary between 20–80%, depending on the study, the population, vaccine strains match, and the outcome measured.^1^ For individuals 60 years of age and older living in the community, influenza vaccination has been shown to be effective against hospitalization from influenza and/or pneumonia and all-cause mortality, but not effective against influenza and influenza-like disease or pneumonia.^2,3^ A major cause of death in influenza pandemics is secondary bacterial infections, especially those due to *Streptococcus pneumonia*, and some of these infections can be prevented with pneumococcal vaccination.^4,5^

The 23-valent pneumococcal polysaccharide vaccine (PPV23) has been approved and licensed in the United States (US) since 1983, and is currently recommended for all adults aged ≥ 65 years.^6^ A vaccine effectiveness rate of 45% against community-acquired pneumococcal pneumonia has been shown in the elderly (mean age, 74.5 years) who received PPV23 within the prior 5 years.^7^ Furthermore, a systematic review concluded that PPV23 vaccination of individuals more than 65 years of age can be cost-effective for the prevention of invasive pneumococcal disease.^8^ The second vaccine, the 13-valent pneumococcal conjugate vaccine (PCV13), which has been used among children since 2010, was approved by FDA in December 2011 for use in those aged ≥ 50 years.^9^ A randomized placebo-controlled trial (CAPiTA trial) was conducted in the Netherlands, and verified the clinical benefit of PCV13 in the prevention of vaccine-type community-acquired pneumonia (46%), nonbacteremic/noninvasive pneumococcal pneumonia (45%), and invasive pneumococcal disease (75%) among adults aged ≥ 65 years.^10,11^ The report of CAPiTA trial was presented to the US Advisory Committee on Immunization Practices (ACIP) in June 2014, which subsequently recommended sequential PCV13 and PPV23 vaccination in August, 2014 for adults ≥ 65 years of age.^12^ Because of the overall decline in the incidence of PCV13 serotype disease following the introduction of PCV13 vaccination of children in 2010, the cost effectiveness of PPV23 vaccination in older adults will inevitably decrease.^13^ However, analyses conducted taking into consideration the indirect effects of pediatric programs have shown that adult age- and risk-based recommendations for PCV13 are still expected to be cost effective.^14^

Since the effectiveness of pneumococcal vaccination had been well-documented, the protective effect of dual influenza and pneumococcal vaccination has been investigated by many research groups. Study has suggested that the concomitant administration of PPV23 and influenza vaccine may have a greater protective effect against pneumonia and influenza in the elderly than the administration of either vaccination alone.^15-19^ Vaccination with both vaccines has been shown to reduce the risk of hospitalization more than if only one or the other was administered.^18,20^ Pneumococcal vaccination has also been shown to reduce specific- and all-cause mortality in individuals more than 60 years of age who have received influenza vaccination.^21^ A 2012 review by Gilchrist et al.^16^ reported that 8 of 9 clinical studies identified found that concomitant influenza and pneumococcal vaccination conferred clinical benefits.

However, whether pneumococcal vaccination should be administered to the elderly as a supplement to influenza vaccination is still debatable. A previous systematic review, which included articles published between 1966–2002, concluded that there is insufficient evidence demonstrating a benefit of pneumococcal vaccination as a supplement to influenza vaccination in the elderly.^22^ A prior study also found that the pneumococcal polysaccharide vaccine did not provide additional protection from pneumonia in the elderly, although it did reduce the incidence of bacteremia.^23^ Furthermore, while an additive effect of dual pneumococcal and influenza vaccination on infectious acute exacerbations was seen in the first year after vaccination, the effect did not persist into the second year and was only significant in patients with chronic respiratory diseases such as chronic obstructive pulmonary disease (COPD).^24^

There was large variation between studies published from 1966 to 2002 with respect to measures including the valency of the vaccines, outcome measures, and duration of follow-up.^22^ In addition, several additional studies regarding influenza plus pneumococcal dual vaccination have been published since 2002. Because the studies published after 2002 still exhibited heterogeneity with respect to study design (either clinical trials or observational studies), definition of vaccination status, and vaccine status ascertainment, an updated meta-analysis was performed to address this important issue in public health. We specifically compared the effectiveness of influenza vaccination alone *vs.* influenza plus pneumococcal dual vaccination for the prevention of pneumonia and mortality in adults ≥ 65 years of age.

## Results

### Literature search

**Figure 1.**
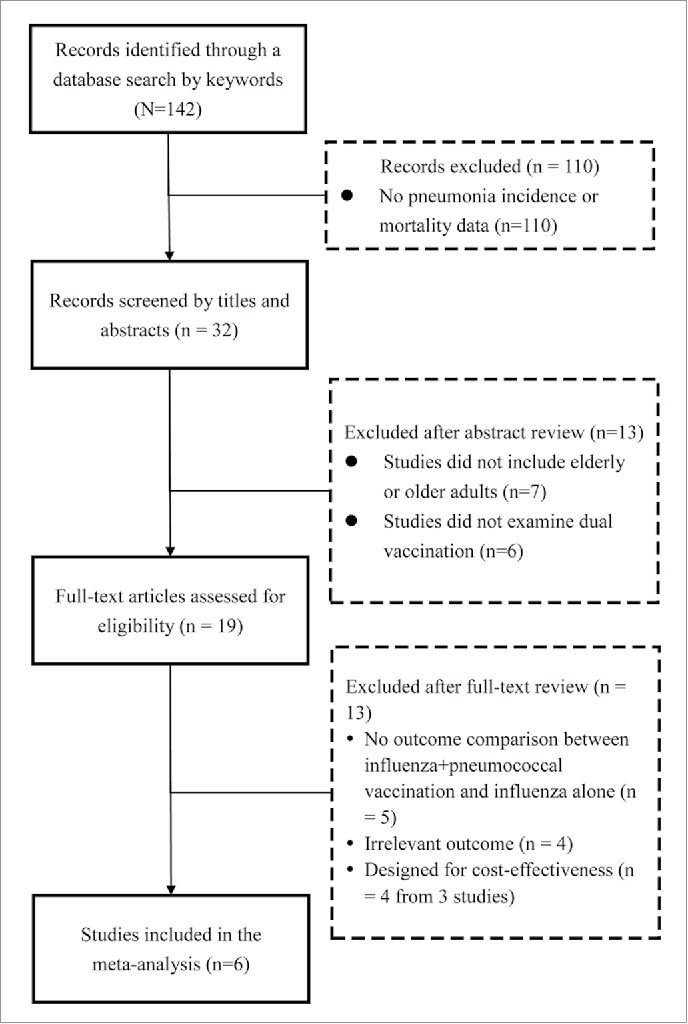
Flow diagram of study selection.

A flow diagram of study selection is shown in Figure 1. Of 142 studies identified in the database searches, 110 were excluded because they did not provide pneumonia incidence or mortality data. Of the remaining 32 articles, 12 were excluded as they did not include adults ≥ 65 years of age (n = 6) or they did not examine dual vaccination (n = 6). The full texts of the remaining 20 articles were then examined, and 14 were excluded (Fig. 1). Thus, 6 studies,^23,25-29^ were included in the systematic review, including 1 randomized controlled trial (RCT), 3 prospective studies, and 2 retrospective studies. The characteristics of the 6 studies are summarized in Table 1, and outcomes are summarized in Table 2. The number of patients that received the influenza vaccination alone ranged from 211 to 29,346 (total = 53,107), and the number that received an influenza+pneumococcal vaccination ranged from 246 to 72,107 (total = 102,068).

**Table 1.** Characteristics of studies included in the meta-analysis.

1st author (publication year)	Study design	Study period	Number of patients	Intervention	Age (y)	Male	COPD	Asthma
Chan (2012)^21^	Prospective cohort	December 2009 to November 2010	246	FV+PV	85.7 ± 7.6	40%	10.20%	n/a
		(12 months)	211	FV	86.0 ± 8.0	34%	12.70%	n/a
Chang (2012)^22^	Retrospective cohort	December 2008 through March 2009 (influenza season)	8142	FV+PV	80.1 ± 4.2	45%	n/a	n/a
		(4 months)	8142	FV	80.1 ± 4.3	46%	n/a	n/a
Kawakami (2010)^23^	RCT	Enrollment during October to November 2005; 2 years (24 months) of follow up	391	FV+PV	78.5 ± 7.3	38%	n/a	n/a
			387	FV	77.7 ± 7.2	32%	n/a	n/a
Hung (2010)^25^	Prospective study	December 3, 2007 to March 31, 2009	7292	FV+PV	77 (71–83)^a^	40%	4.40%	2.20%
		(16 months)	2076	FV	75 (70–80)^a^	45%	4.60%	2.20%
Christenson (2004)^24^	Prospective study	December 1999 to November 2000; 1 year (12 months) of follow up.	72107	FV+PV	≥65 ^b^	n/a	n/a	n/a
			29346	FV				
Honkanen (1999)^19^	Retrospective	Cohort I: start in November 30, 1992; Chort II: start in November 15, 1993; followed until December 31, 1994 (13 months) for pneumonia, and December 31, 1995 (25 months) for bacteremia	13980	FV+PV	Cohort I: 74.1 ± 6.8 Cohort II: 72.8 ± 6.5	38%	6.10%	
			12,945	FV	Cohort I: 73.9 ± 7.0 Cohort II: 73.6 ± 6.5	38%	6.30%	

COPD, chronic obstructive pulmonary disease; FV, influenza vaccination; n/a, not available; PV, pneumococcal vaccination; RCT, randomized controlled trial.

a Median (range).

b No mean age was reported, and the population was stratified by age.

**Table 2.** Outcomes of studies included in the meta-analysis.

			All-cause pneumonia^a^		All-cause mortality	
1st Author (publication year)	Intervention	Vaccine status and ascertainment	Incidence(per person years)^b^	RR (95% CI)^c^	N (%)	RR (95% CI)^c^
Chan (2012)	FV+PV	Record of nursing home showed the vaccination status of each resident.	n/a	n/a	42 (17.1)	FV+PV vs. FV: aHR = 0.54 (0.35–0.84)
	FV		n/a	n/a	57 (27)	
Chang (2012)	FV+PV	Record of National Health Insurance showed re-imbursement of influenza vaccination during the free influenza vaccine period.	2.1% (0.021 per person years)	FV+PV vs. FV: aOR = 0.85 (0.69–1.05)	1.30%	FV+PV vs. FV: aOR = 0.74 (0.57–0.96)
	FV		2.4% (0.024 per person years)		1.70%	
Kawakami (2010)	FV+PV	In this RCT, the participants received seasonal influenza vaccine in 2005, and received 23-valent PV in 1-month interval after FV in FV plus PV group.	8.6% (0.086 per person year)	FV+PV vs. FV: aHR = 0.73 (0.44–1.23)	23 (5.9)	n/a
	FV		10.5% (0.105 per person year)		25 (6.5)	
Hung (2010)	FV+PV	In this prospective study, all participants except the 23-valent PV-alone group and the unvaccinated group were received intramuscular 2007–2008 and 2008–2009 trivalent FV.	7.3% (0.073 per person years)	FV+PV vs. FV: aHR = 0.76 (0.62–0.93)	n/a	Vaccinated vs. unvaccinated: aHR = 0.65 (0.55–0.77) FV+PV vs. FV: aHR = 0.86 (0.64–1.16)
	FV		9.5% (0.095 per person years)		n/a	Vaccinated vs. unvaccinated: HR = 0.78 (0.61–1.0)
Christenson (2004)	FV+PV	In this prospective study, participants received trivalent FV alone, 23-valent PV or both FV and PV.	1.6% (0.016 per person years)	Vaccinated vs. unvaccinated: aOR = 0.71 (0.65–0.75) FV+PV vs. FV: aOR = 0.756 (0.676–0.844)	n/a	Vaccinated vs. unvaccinated: aOR = 0.29 (0.06–1.31) FV+PV vs. FV: aOR = 0.414 (0.047–3.64)
	FV		2.14% (0.0214 per person years)	Vaccinated vs. unvaccinated: aOR = 0.94 (0.86–1.02)	n/a	Vaccinated vs. unvaccinated: aOR = 0.70 (0.15–3.21)
Honkanen (1999)	FV+PV	Records from local health centers in 23 administrative districts in northern Finland (cohort I) showed trivalent FV and 23-valent PV or trivalent FV alone in autumn 1992, and this was extended to a further 12 districts (cohort II) in 1993.	0.74% (0.0074 per person years)	FV+PV vs. FV: Risk ratio = 1.2 (0.9–1.5)^d^	n/a	n/a
	FV		0.63% (0.0063 per person years)		n/a	

FV, influenza vaccination; PV, pneumococcal vaccination; RR, relative risk; aHR, adjusted hazard ratio; aOR, adjust odds ratio; n/a, not available.

a Pneumonia refer to ICD-9-CM: 480–486 or ICD-10-CM: J12–18.

b The incidence of all-cause pneumonia were converted to per person years for all studies.

c A0 RR > 1 indicates that influenza + pneumococcal vaccination is associated with a higher pneumonia or all-cause mortality rate than influenza vaccination alone, whereas an RR < 1 indicates that dual vaccination is associated with a lower pneumonia or all-cause mortality rate than influenza vaccination alone. A RR = 1 indicates the rates are similar between the 2 treatment groups.

d The definition of RR in Honkanen et al.^19^ was irrelevant to either OR or HR. The RR in this study was calculated based on the ratio between outcome rates per 1,000 person-years of the influenza+pneumococcal group and the influenza alone group.

### Pneumonia rate

**Figure 2.**
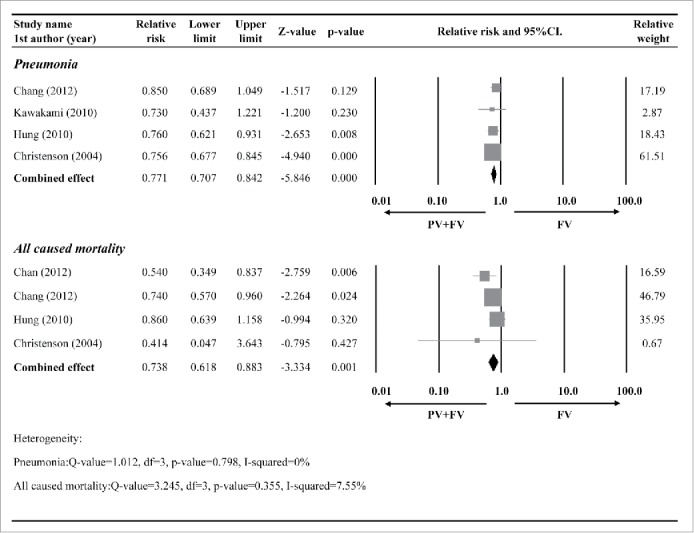
Forest plots comparing the pneumonia and all-cause mortality rates between patients that that received the influenza vaccination (FV) alone and those that received an influenza plus pneumococcal vaccination (PV). Abbreviations: RR, relative risk; CI, confidence interval; Lower limit, lower boundary of the 95% CI; Upper limit, upper boundary of the 95% CI.

Four studies^26-29^ provided complete data regarding the incidence of pneumonia between patients that received the influenza vaccination alone and those that received influenza+pneumococcal vaccination. The study by Honkanen et al.^23^ was excluded from the meta-analysis since the definition of relative risk (RR) in this report was irrelevant to either the odds ratio (OR) or hazard ratio (HR). In that study, RR was calculated based on the ratio between outcome rates per 1000 person years of the influenza+pneumococcal group and the influenza alone group.^23^ There was no evidence of heterogeneity among the 4 included studies (Q statistic = 1.012, I^2^ = 0%, *P* = 0.798); thus, a fixed-effects model of analysis was used. The analysis indicated that influenza+pneumococcal vaccination was associated with a significantly lower pneumonia rate than influenza vaccination alone (RR = 0.738, 95% confidence interval [CI]: 0.618–0.883, *P* = 0.001) (Fig. 2).

### All-cause mortality

Four studies^23,26,28,29^ provided complete data regarding the all-cause mortality rate between patients that received influenza vaccination alone and those that received influenza+pneumococcal vaccination. There was no evidence of heterogeneity among the 4 studies (Q statistic = 3.245, I^2^ = 7.55%, *P* = 0.355); thus, a fixed-effects model of analysis was used. The analysis indicated that influenza+pneumococcal vaccination was associated with a significantly lower all-cause mortality rate than influenza vaccination alone (RR = 0.738, 95% CI: 0.618–0.883, *P* = 0.001) (Fig. 2)

### Sensitivity analysis

**Figure 3.**
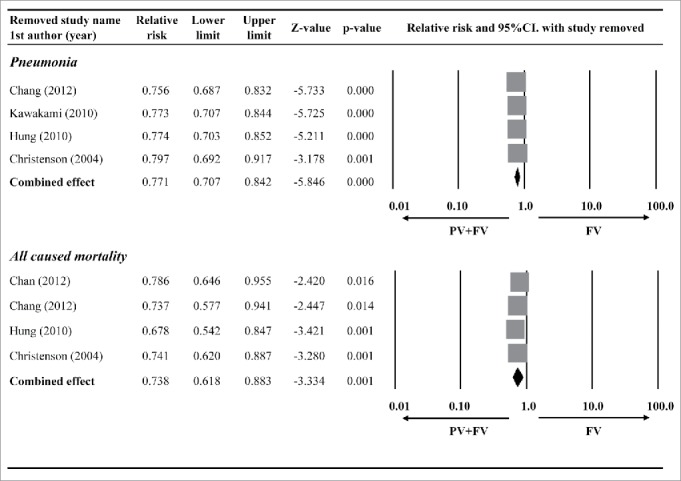
Sensitivity analysis using the leave-one-out approach of the pneumonia and all-cause mortality rates between patients that that received the influenza vaccination (FV) alone and those that received an influenza plus pneumococcal vaccination (PV). Abbreviations: RR, relative risk; CI, confidence interval; Lower limit, lower boundary of the 95% CI; Upper limit, upper boundary of the 95% CI.

Sensitivity analyses revealed that the direction and magnitude of the combined estimates did not change markedly with the exclusion of individual studies (Fig. 3). This indicates that the reliability of the meta-analysis was good.

### Quality assessment

Study quality was assessed according to The Newcastle-Ottawa Scale coding manual for cohort studies, with scores ranging from 0–10 and higher scores indicating better quality.^30^ Two of the studies achieved scores of 7, and 4 studies achieved scores of 8, indicating that the overall quality of the studies was good.

## Discussion

The results of this meta-analysis support the concomitant vaccination elderly persons with influenza and pneumococcal vaccines. Dual vaccination was associated with a lower incidence of pneumonia and a lower all-cause mortality rate in this group of patients. The additive benefits of influenza and pneumococcal vaccination during influenza season among elderly persons with chronic lung disease had been reported by Nichol in 1999.^17^ She showed that influenza vaccination alone resulted in a 52% reduction in hospitalization for pneumonia and 70% reduction in death, and PPV23 vaccination alone reduce the rate of hospitalization for pneumonia and mortality by 27% and 34%, respectively. However, when both vaccines were given concomitantly, the reductions in the pneumonia hospitalization and death rates were greater than when either vaccine was given alone (63% and 81%, respectively). Several large-scale studies also support a dual vaccination strategy. A multicenter case-controlled study in 36 Spanish hospitals conducted during the 2009–2010 pandemic and 2010–2011 influenza epidemic assessed the effectiveness of the pneumococcal polysaccharide vaccine alone and in combination with influenza vaccination for preventing influenza hospitalization.^20^ The adjusted effectiveness of dual PPV23 and influenza vaccination was 81% in all patients and 76% in patients ≥ 65 years old, while the adjusted effectiveness of influenza vaccination alone was only 58%. A study of Chinese older adults in a nursing home showed that dual influenza and pneumococcal vaccination was more effective than the influenza vaccination alone in reducing all-cause mortality, mortality attributable to pneumonia, and mortality attributable to vascular causes.^25^ However, the correlation between clinical efficacy and immune response induced by dual vaccination in the elderly is still debatable. Grille et al.^19^ studied antibody response in elderly individuals who received influenza and pneumococcal vaccines administered alone or in combination. No differences in the percentage of protected individuals or the percentage of subjects who seroconverted were observed between those that received the influenza vaccine alone and those that received influenza and pneumococcal vaccines. Simultaneous immunization with pneumococcal and influenza vaccines has not been associated with an increased rate of adverse reactions (e.g. fever, headache, pain, local swelling) compared with administration of pneumococcal vaccine alone.^31^ Similarly, Socan et al.^32^ reported that simultaneous immunization with pneumococcal and influenza vaccines was not associated with an increased rate of complaints compared with administration of pneumococcal vaccine alone.

Two adult vaccines to prevent pneumococcal disease have been approved in the US since 1983. PPV23 is currently recommended for all adults aged ≥ 65 years. The second vaccine, PCV13, was approved by the US FDA in December 2011 for use in those aged ≥ 50 years.^9^ However, the Centers for Disease Control (CDC) in the US has not made recommendations for PCV13 use in adults that do not have immunocompromising conditions, owing to a number of scientific and public health uncertainties. A randomized placebo-controlled trial (CAPiTA trial) conducted in the Netherlands verified the clinical benefit of PCV13 in the prevention of vaccine-type community-acquired pneumonia (46%), nonbacteremic/noninvasive pneumococcal pneumonia (45%), and invasive pneumococcal disease (75%) among adults aged ≥ 65 years.^11^ The report of CAPiTA trial was presented to the US Advisory Committee on Immunization Practices (ACIP) in June 2014, which subsequently recommended sequential PCV13 and 23-valent pneumococcal polysaccharide vaccination for adults ≥ 65 years in August, 2014.^10,12^ Several RCTs conducted in the US and Europe among older adults showed that PCV13 induced an immune response as good as or better than that induced by PPSV23, and that both have similar safety profiles.^33,34^

While study has demonstrated that pneumococcal vaccination is cost-effective in adults and the elderly,^8^ few studies have compared the cost effectiveness of influenza plus pneumococcal vaccination with influenza vaccination alone. Cia et al.^35^ studied dual vaccination in elderly persons in Japan, and found that for every 100,000 people over 65 years of age the cost effectiveness ratio of influenza only vaccination was 516,332 Japanese yen (approximate 4,800 USD) per 1 year of life saved while for combined influenza and pneumococcal vaccination the ratio was 459,874 Japanese yen (approximate 4,300 USD) for the same benefit. In a similar study of the elderly living in long-term care facilities in Hong Kong, You et al.^36^ showed that combined influenza and pneumococcal vaccination was more likely to result in higher quality-adjusted life years and lower total cost than influenza vaccination alone. Herd immunity (indirect effects) is a form of indirect protection from infectious disease that occurs when a large percentage of a population has become immune to an infection, thereby providing a measure of protection for individuals who are not immune.^37^ Earlier studies before pneumococcal conjugate vaccine (PCV) vaccination of children showed that PPV23 vaccination was cost effective when administered with influenza vaccine.^38^ After PCV7 and PCV13 vaccinations of children were performed, it resulted in a similar decrease in PCV7 and PCV13 serotype invasive pneumococcal disease in older individuals due to herd immunity. Because of the overall decline in the incidence of PCV13 serotype disease, the cost effectiveness of PPV23 vaccination in older adults will inevitably decrease.^39^ In fact, in European countries, pneumococcal vaccination to prevent invasive pneumococcal disease in elderly adults has been very cost effective.^40^

A number of studies that suggested dual vaccination is more protective than vaccination with only one vaccine were not included in the analysis.^15-20^ In 2 studies^15,17^ there was no comparison data for influenza+pneumococcal and influenza alone, 1 study was a systematic review,^16^ 2 studies did not report any outcomes of interest,^18,19^ and 1 study was not designed for to evaluate the elderly population.^20^ The systematic review by Gilchrist et al.,^16^ which did not include a meta-analysis, included a total of 9 studies: 2 were RCTs and the remainder were observational studies. Of the 7 observational studies, 4 were cohort studies in the elderly, 2 were cohort studies in the elderly with chronic illness, and 1 was a cohort study in HIV-infected participants. Although we also included RCTs, 2-arm prospective studies, and retrospective cohort studies, a meta-analysis was conducted rather than only a systematic review. In addition, we only included studies designed to examine influenza vaccination alone versus dual pneumococcal+influenza vaccination and outcomes included the incidence of pneumonia, length of hospital stay, and overall mortality rate.

The primary limitation of the current analysis is the limited number of studies. While this is in part a result of the inclusion and exclusion criteria, it also reflects the limited number of studies examining this topic, especially studies designed for comparison between concomitant pneumococcal and influenza vaccination (dual vaccination) vs. influenza vaccination alone (single vaccination). The included studies also differed in design, i.e., 1 RCT, prospective and retrospective studies. Heterogeneity in the definition of vaccine status and ascertainment (summarized in Table 2) is another limitation of study. In addition, the models used to estimate the risk of pneumonia and mortality were diverse among the studies. Because OR and HR were used inconsistently in the various analyses, the term “risk” may refer to OR or HR.^41^ Previous meta-analyses in the literature also considered HR and OR as RR.^42,43^ Another potential limitation of this study is that it cannot account for the ‘healthy user’ effect in the individual studies, since elderly usually suffer from various chronic diseases. The number of person-years included in each influenza+pneumococcal group is important. Fedson et al.^39^ has argued that none of the meta-analyses of this topic have included an adequate number of person-years of observation to rule out false negative results. We realize that the length of follow-up is diverse among the included studies, and ranged from 4 months^26^ to 24 months.^27^ Although the incidence rate of pneumonia has been converted to per person year from original data for each study (Table 2), we cannot rule out false negative results due to the nature of a meta-analysis. There is a similar problem with the analysis of mortality rate, and this is also considered a limitation of the study.

In conclusion, the results of this study support concomitant pneumococcal and influenza vaccination of the elderly. A dual vaccination strategy is associated with lower pneumonia and mortality rates. Sequential PCV13 and PPV23 vaccination for adults ≥ 65 years has been recommended by ACIP of US FDA. Though the findings of this study are promising, the value of concomitant pneumococcal and influenza vaccination of the elderly needs to be confirmed by large scale clinical trials.

## Material and methods

### Literature search strategy

This study was conducted in accordance with Preferred Reporting Items for Systematic Reviews and Meta-Analyses (PRISMA) guidelines.^44^ Medline, Cochrane, CENTRAL, EMBASE, and Google Scholar databases were searched through June 30, 2015 using combinations of the keywords: pneumococcal, influenza, pneumococcal, pneumonia, vaccination, vaccine, immunization, effectiveness, mortality, elderly, old adults. Reference lists from relevant studies were also examined to identify relevant studies. The searches were limited to English language articles. Searches were conducted by 2 independent reviewers, and a third was consulted for resolution of any disagreements.

### Study selection criteria

Inclusion criteria were: 1) Randomized controlled trials (RCTs), 2-arm prospective studies, or retrospective cohort studies; 2) Patients were ≥ 65 years of age with or without chronic respiratory disease; 3) Patients received the influenza vaccine alone or dual pneumococcal and influenza vaccination; 4) Results included incidence of pneumonia (ICD-9-CM: 480–486 or ICD-10-CM: J12–18), length of hospital stay, and overall mortality rate. Letters, comments, editorials, and case reports were excluded. In addition, studies not designed to compare influenza plus pneumococcal dual vaccination vs. influenza vaccination alone, and those designed to examine cost effectiveness were excluded.

### Quality assessment

The Newcastle-Ottawa Scale was used for assessing the quality of nonrandomized studies in meta-analysis.^30^ Briefly, the instrument scores the following categories: (i) random sequence generation; (ii) allocation concealment; (iii) blinding of patients, personnel, and assessor; (iv) adequate assessment of each outcome; (v) avoidance of a selective outcome report; and (vi) presence or absence of an intention-to-treat analysis. The maximum possible score is 10, with higher scores indicating higher quality. Scoring was performed by 2 independent reviewers, and a third reviewer was consulted for resolution of any disagreements.

### Data extraction

The following data was extracted from studies that met the inclusion criteria: first author's name, publication year, study design and period, patient demographic data (e.g., age, gender), comorbidities (e.g., COPD, asthma, respiratory infectious diseases), adjusted odd ratio (aOR) and/or adjusted hazard ratio (aHR) and risk ratio relating to the incidence of recurrent respiratory tract infections (e.g., pneumococcal pneumonia, overall pneumonia rate), length of hospital stay, and all-cause mortality rate.

### Endpoints and data analysis

The outcomes of the meta-analysis were incidence of pneumonia and all-cause mortality rate for patients that received the influenza vaccination alone as compared to those that received influenza plus pneumococcal dual vaccination. The combined effect RR, including aHR and aOR with corresponding 95% confidence interval (CIs) for the 2 events were calculated for the 2 groups of patients. Definitions of vaccination status and vaccine status ascertainment were based on the Protocol for case-control studies of influenza vaccine effectiveness in the European Union and European Economic Area Member States.^45^ Statistical significance was indicated by a 2-sided *P* value < 0.05.

The Cochran's Q and I^2^ statistics were used to examine homogeneity of the included articles. The percentage of the total variability in effect estimates among trials due to heterogeneity rather than chance is indicated by the value of the I^2^ statistic. Random-effects models of analysis were used if heterogeneity was detected (Cochran Q *P* < 0.10 or I^2^ > 50%). Otherwise, fixed-effects models were used. Sensitivity analysis was performed using the leaveone-out approach. If there were < 10 studies, publication bias analysis was not assessed because ≥ 10 studies are needed to detect funnel plot asymmetry.^46^ All analyses were performed using Comprehensive Meta-Analysis statistical software, version 2.0 (Biostat, Englewood, NJ). The Indirect Treatment Comparison (ITC) computer program, version 1.0 was applied to transfer the data from indirect comparison to direct comparisons.^47^

## References

[1] World Health Organization, Strategic Advisory Group of Experts (SAGE) Working Group Background paper on influenza vaccines and immunization. 2012 Available at: http://www.who.int/entity/immunization/sage/meetings/2012/april/1_Background_Paper_Mar26_v13_cleaned.pdf?ua=1. Accessed: January 5, 2016.

[2] Jefferson T, Rivetti D, Rivetti A, Rudin M, Di Pietrantonj C, Demicheli V. Efficacy and effectiveness of influenza vaccines in elderly people: a systematic review. Lancet 2005; 366:1165-74; PMID:16198765; http://dx.doi.org/10.1016/S0140-6736(05)67339-4 16198765

[3] Darvishian M, Gefenaite G, Turner RM, Pechlivanoglou P, Van der Hoek W, Van den Heuvel ER, Hak E. After adjusting for bias in meta-analysis seasonal influenza vaccine remains effective in community-dwelling elderly. J Clin Epidemiol 2014; 67:734-44; PMID:24768004; http://dx.doi.org/10.1016/j.jclinepi.2014.02.009 24768004

[4] Palacios G, Hornig M, Cisterna D, Savji N, Bussetti AV, Kapoor V, Hui J, Tokarz R, Briese T, Baumeister E, et al. Streptococcus pneumoniae coinfection is correlated with the severity of H1N1 pandemic influenza. PLoS One 2009; 4:e8540; PMID:20046873; http://dx.doi.org/10.1371/journal.pone.0008540 20046873 PMC2795195

[5] Walter ND, Taylor TH, Shay DK, Thompson WW, Brammer L, Dowell SF, Moore MR, Active Bacterial Core Surveillance Team. Influenza circulation and the burden of invasive pneumococcal pneumonia during a non-pandemic period in the United States. Clin Infect Dis 2010; 50:175-83; PMID:20014948; http://dx.doi.org/10.1086/649208 20014948

[6] Centers for Disease Control and Prevention (CDC). Updated recommendations for prevention of invasive pneumococcal disease among adults using the 23-valent pneumococcal polysaccharide vaccine (PPSV23). MMWR Morb Mortal Wkly Rep 2010; 59:1102-6; PMID:20814406 20814406

[7] Ochoa-Gondar O, Vila-Corcoles A, Rodriguez-Blanco T, Gomez-Bertomeu F, Figuerola-Massana E, Raga-Luria X, Hospital-Guardiola I. Effectiveness of the 23-valent pneumococcal polysaccharide vaccine against community-acquired pneumonia in the general population aged ≥ 60 years: 3 years of follow-up in the CAPAMIS study. Clin Infect Dis 2014; 58:909-17; PMID:24532544; http://dx.doi.org/10.1093/cid/ciu002 24532544

[8] Ogilvie I, Khoury AE, Cui Y, Dasbach E, Grabenstein JD, Goetghebeur M. Cost-effectiveness of pneumococcal polysaccharide vaccination in adults: a systematic review of conclusions and assumptions. Vaccine 2009; 27:4891-904; PMID:19520205; http://dx.doi.org/10.1016/j.vaccine.2009.05.061 19520205

[9] Smith KJ, Wateska AR, Nowalk MP, Raymund M, Lee BY, Zimmerman RK. Modeling of cost effectiveness of pneumococcal conjugate vaccination strategies in U.S. older adults. Am J Prev Med 2013; 44:373-81; PMID:23498103; http://dx.doi.org/10.1016/j.amepre.2012.11.035 23498103 PMC3601581

[10] Isturiz R, Webber C. Prevention of adult pneumococcal pneumonia with the 13-valent pneumococcal conjugate vaccine: CAPiTA, the community-acquired pneumonia immunization trial in adults. Hum Vaccin Immunother 2015; 11:1825-7; PMID:26076136; http://dx.doi.org/10.1080/21645515.2015.1043502 26076136 PMC4514202

[11] Bonten MJ, Huijts SM, Bolkenbaas M, Webber C, Patterson S, Gault S, van Werkhoven CH, van Deursen AM, Sanders EA, Verheij TJ, et al. Polysaccharide conjugate vaccine against pneumococcal pneumonia in adults. N Engl J Med 2015; 372:1114-25; PMID:25785969; http://dx.doi.org/10.1056/NEJMoa1408544 25785969

[12] Tomczyk S, Bennett NM, Stoecker C, Gierke R, Moore MR, Whitney CG, Hadler S, Pilishvili T, Centers for Disease Control and Prevention (CDC) Use of 13-valent pneumococcal conjugate vaccine and 23-valent pneumococcal polysaccharide vaccine among adults aged ≥ 65 years: recommendations of the Advisory Committee on Immunization Practices (ACIP). MWR Morb Mortal Wkly Rep 2014; 63:822-5 PMC5779453 25233284

[13] Fedson DS, Liss C. Precise answers to the wrong question: prospective clinical trials and the meta-analyses of pneumococcal vaccine in elderly and high-risk adults. Vaccine 2004; 22:927-46; PMID:15161070; http://dx.doi.org/10.1016/j.vaccine.2003.09.027 15161070

[14] Hollingsworth R, Isturiz R. Pneumococcal vaccination of older adults: conjugate or polysaccharide? Hum Vaccin Immunother 2014; 10:45-6; PMID:24018552; http://dx.doi.org/10.4161/hv.26330 24018552 PMC4181019

[15] Li C, Gubbins PO, Chen GJ. Prior pneumococcal and influenza vaccinations and in-hospital outcomes for community-acquired pneumonia in elderly veterans. J Hosp Med 2015; 10:287-93; PMID:25676363; http://dx.doi.org/10.1002/jhm.2328 25676363

[16] Gilchrist SA, Nanni A, Levine O. Benefits and effectiveness of administering pneumococcal polysaccharide vaccine with seasonal influenza vaccine: an approach for policymakers. Am J Public Health 2012; 102:596-605; PMID:22397339; http://dx.doi.org/10.2105/AJPH.2011.300512 22397339 PMC3489371

[17] Nichol KL. The additive benefits of influenza and pneumococcal vaccinations during influenza seasons among elderly persons with chronic lung disease. Vaccine 1999; 17:S91-3; PMID:10471189; http://dx.doi.org/10.1016/S0264-410X(99)00114-0 10471189

[18] Christenson B, Pauksen K, Sylvan SP. Effect of influenza and pneumococcal vaccines in elderly persons in years of low influenza activity. Virol J 2008; 5:52; PMID:18442371; http://dx.doi.org/10.1186/1743-422X-5-52 18442371 PMC2390520

[19] Grilli G, Fuiano L, Biasio LR, Pregliasco F, Plebani A, Leibovitz M, Ugazio AG, Vacca F, Profeta ML. Simultaneous influenza and pneumococcal vaccination in elderly individuals. Eur J Epidemiol 1997; 13:287-91; PMID:9258527; http://dx.doi.org/10.1023/A:1007398606807 9258527

[20] Domínguez A, Castilla J, Godoy P, Delgado-Rodríguez M, Saez M, Soldevila N, Astray J, Mayoral JM, Martín V, Quintana JM, et al. Effectiveness of vaccination with 23-valent pneumococcal polysaccharide vaccine in preventing hospitalization with laboratory confirmed influenza during the 2009–2010 and 2010–2011 seasons. Hum Vaccin Immunother 2013; 9:865-73; http://dx.doi.org/10.4161/hv.23090 23563516 PMC3903906

[21] Vila-Corcoles A, Ochoa-Gondar O, Rodriguez-Blanco T, de Diego-Cabanes C, Satue-Gracia E, Vila-Rovira A, Torrente Fraga C, EPIVAC Research Group. Evaluating clinical effectiveness of pneumococcal vaccination in preventing stroke: the CAPAMIS Study, 3-year follow-up. J Stroke Cerebrovasc Dis 2014; 23:1577-84; PMID:24656243; http://dx.doi.org/10.1016/j.jstrokecerebrovasdis.2013.12.047 24656243

[22] Assendelf WJ, Scholten RJ, Offringa M. Pneumococcal vaccination for the elderly in The Netherlands? Assessment of the quality and content of available comparative studies. Neth J Med 2004; 62:36-44; PMID:15127829 15127829

[23] Honkanen PO, Keistinen T, Miettinen L, Herva E, Sankilampi U, Läärä E, Leinonen M, Kivelä SL, Mäkelä PH. Incremental effectiveness of pneumococcal vaccine on simultaneously administered influenza vaccine in preventing pneumonia and pneumococcal pneumonia among persons aged 65 years or older. Vaccine 1999; 17:2493-500; PMID:10418894; http://dx.doi.org/10.1016/S0264-410X(99)00069-9 10418894

[24] Furumoto A, Ohkusa Y, Chen M, Kawakami K, Masaki H, Sueyasu Y, Iwanaga T, Aizawa H, Nagatake T, Oishi K. Additive effect of pneumococcal vaccine and influenza vaccine on acute exacerbation in patients with chronic lung disease. Vaccine 2008; 26:4284-9; PMID:18585831; http://dx.doi.org/10.1016/j.vaccine.2008.05.037 18585831

[25] Chan TC, Hung IF, Luk JK, Shea YF, Chan FH, Woo PC, Chu LW. Prevention of mortality and pneumonia among nursing home older adults by dual pneumococcal and seasonal influenza vaccination during a pandemic caused by novel pandemic influenza A (H1N1). J Am Med Dir Assoc 2012; 13:698-703; PMID:22722051; http://dx.doi.org/10.1016/j.jamda.2012.05.009 22722051

[26] Chang YC, Chou YJ, Liu JY, Yeh TF, Huang N. Additive benefits of pneumococcal and influenza vaccines among elderly persons aged 75 years or older in Taiwan–a representative population-based comparative study. J Infect 2012; 65:231-8; PMID:22561486; http://dx.doi.org/10.1016/j.jinf.2012.04.014 22561486

[27] Kawakami K, Ohkusa Y, Kuroki R, Tanaka T, Koyama K, Harada Y, Iwanaga K, Yamaryo T, Oishi K. Effectiveness of pneumococcal polysaccharide vaccine against pneumonia and cost analysis for the elderly who receive seasonal influenza vaccine in Japan. Vaccine 2010; 28:7063-9; PMID:20723631; http://dx.doi.org/10.1016/j.vaccine.2010.08.010 20723631

[28] Christenson B, Hedlund J, Lundbergh P, Ortqvist A. Additive preventive effect of influenza and pneumococcal vaccines in elderly persons. Eur Respir J 2004; 23:363-8; PMID:15065822; http://dx.doi.org/10.1183/09031936.04.00063504 15065822

[29] Hung IF, Leung AY, Chu DW, Leung D, Cheung T, Chan CK, Lam CL, Liu SH, Chu CM, Ho PL, et al. Prevention of acute myocardial infarction and stroke among elderly persons by dual pneumococcaland influenza vaccination: a prospective cohort study. Clin Infect Dis 2010; 51:1007-16; PMID:20887208; http://dx.doi.org/10.1086/656587 20887208

[30] Wells G, Shea B, O'Connell J, Robertson J, Peterson V, Welch V, Losos M, Tugwell P The Newcastle-Ottawa scale (NOS) for assessing the quality of nonrandomised studies in meta-analysis. Available at: http://www.ohri.ca/programs/clinical_epidemiology/nos_manual.pdf

[31] Frenck RW Jr, Gurtman A, Rubino J, Smith W, van Cleeff M, Jayawardene D, Giardina PC, Emini EA, Gruber WC, Scott DA, et al. Randomized, controlled trial of a 13-valent pneumococcal conjugate vaccine administered concomitantly with an influenza vaccine in healthy adults. Clin Vaccine Immunol 2012; 19:1296-303; PMID:22739693; http://dx.doi.org/10.1128/CVI.00176-12 22739693 PMC3416075

[32] Socan M, Frelih T, Janet E, Petras T, Peternelj B. Reactions after pneumococcal vaccine alone or in combination with influenza vaccine. Vaccine 2004; 22:3087-91; PMID:15297059; http://dx.doi.org/10.1016/j.vaccine.2004.02.003 15297059

[33] Shiramoto M, Hanada R, Juergens C, Shoji Y, Yoshida M, Ballan B, Cooper D, Gruber WC, Scott DA, Schmoele-Thoma B. Immunogenicity and safety of the 13-valent pneumococcal conjugate vaccine compared to the 23-valent pneumococcal polysaccharide vaccine in elderly Japanese adults. Hum Vaccin Immunother 2015; 11:2198-206; PMID:26176163; http://dx.doi.org/10.1080/21645515.2015.1030550 26176163 PMC4635730

[34] Jackson LA, Gurtman A, van Cleeff M, Jansen KU, Jayawardene D, Devlin C, Scott DA, Emini EA, Gruber WC, Schmoele-Thoma B. Immunogenicity and safety of a 13-valent pneumococcal conjugate vaccine compared to a 23-valent pneumococcal polysaccharide vaccine in pneumococcal vaccine-naive adults. Vaccine 2013; 31:3577-84; PMID:23688526; http://dx.doi.org/10.1016/j.vaccine.2013.04.085 23688526

[35] Cai L, Uchiyama H, Yanagisawa S, Kamae I. Cost-effectiveness analysis of influenza and pneumococcal vaccinations among elderly people in Japan. Kobe J Med Sci 2006; 52:97-109; PMID:16855372 16855372

[36] You JH, Wong WC, Ip M, Lee NL, Ho SC. Cost-effectiveness analysis of influenza and pneumococcal vaccination for Hong Kong elderly in long-term care facilities. J Epidemiol Community Health 2009; 63:906-11; PMID:19608558; http://dx.doi.org/10.1136/jech.2008.081885 19608558

[37] Smith KJ, Wateska AR, Nowalk MP, Raymund M, Nuorti JP, Zimmerman RK. Cost-effectiveness of adult vaccination strategies using pneumococcal conjugate vaccine compared with pneumococcal polysaccharide vaccine. JAMA 2012; 307:804-12; PMID:22357831 22357831 10.1001/jama.2012.169 PMC3924773

[38] Ament A, Baltussen R, Duru G, Rigaud-Bully C, de Graeve D, Ortqvist A, Jönsson B, Verhaegen J, Gaillat J, Christie P, et al. Cost-effectiveness of pneumococcal vaccination of older people: a study in 5 western European countries. Clin Infect Dis 2000; 31:444-50; PMID:10987703; http://dx.doi.org/10.1086/313977 10987703

[39] Fedson DS, Guppy MJ. Pneumococcal vaccination of older adults: conjugate or polysaccharide? Hum Vaccin Immunother 2013; 9:1382-4; PMID:23732892; http://dx.doi.org/10.4161/hv.24692 23732892 PMC3901836

[40] Evers SM, Ament AJ, Colombo GL, Konradsen HB, Reinert RR, Sauerland D, Wittrup-Jensen K, Loiseau C, Fedson DS. Cost-effectiveness of pneumococcal vaccination for prevention of invasive pneumococcal disease in the elderly: an update for 10 Western European countries. Eur J Clin Microbiol Infect Dis 2007; 26:531-40; PMID:17570001; http://dx.doi.org/10.1007/s10096-007-0327-z 17570001

[41] Raboisson D, Mounié M, Maigné E. Diseases, reproductive performance, and changes in milk production associated with subclinical ketosis in dairy cows: a meta-analysis and review. J Dairy Sci 2014; 97:7547-63; PMID:25306269; http://dx.doi.org/10.3168/jds.2014-8237 25306269

[42] Zheng Y, Lu C, Wei S, Li Y, Long L, Yin P. Association of red blood cell transfusion and in-hospital mortality in patients admitted to the intensive care unit: a systematic review and meta-analysis. Crit Care 2014; 18:515. doi: 10.1186/s13054-014-0515-z; PMID:25394759 25394759 PMC4256753

[43] Larsson SC, Wolk A. Overweight, obesity and risk of liver cancer: a meta-analysis of cohort studies. Br J Cancer 2007; 97:1005-8; PMID:17700568 17700568 10.1038/sj.bjc.6603932 PMC2360408

[44] Liberati A, Altman DG, Tetzlaff J, Mulrow C, Gøtzsche PC, Ioannidis JP, Clarke M, Devereaux PJ, Kleijnen J, Moher D. The PRISMA statement for reporting systematic reviews and meta-analyses of studies that evaluate health care interventions: explanation and elaboration. Ann Intern Med 2009; 151:W65-94; PMID:19622512; http://dx.doi.org/10.7326/0003-4819-151-4-200908180-00136 19622512

[45] Protocol for case-control studies of influenza vaccine effectiveness in the European Union and European Economic Area Member States ECDC Technical Document. Available at: http://ecdc.europa.eu/en/publications/Publications/0907_TED_Influenza_AH1N1_Measuring_Influenza_Vaccine_Effectiveness_Protocol_Case_Control_Studies.pdf. Accessed: 61, 2016

[46] Sterne JA, Sutton AJ, Ioannidis JP, Terrin N, Jones DR, Lau J, Carpenter J, Rücker G, Harbord RM, Schmid CH, et al. Recommendations for examining and interpreting funnel plot asymmetry in meta-analyses of randomised controlled trials. BMJ 2011; 343:d4002; PMID:21784880; http://dx.doi.org/10.1136/bmj.d4002 21784880

[47] Wells GA, Sultan SA, Chen L, Khan M, Coyle D Indirect treatment comparison [computer program]. Version 1.0 Canadian Agency for Drugs and Technologies in Health; Ottawa, 2009

